# Association Between Cancer Prevalence and Different Socioeconomic Strata in the US: The National Health and Nutrition Examination Survey, 1999–2018

**DOI:** 10.3389/fpubh.2022.873805

**Published:** 2022-07-22

**Authors:** Mingsi Wang, Yang Liu, Yi Ma, Yue Li, Chengyao Sun, Yi Cheng, Pengxin Cheng, Guoxiang Liu, Xin Zhang

**Affiliations:** ^1^Department of Health Economics, College of Health Management of Harbin Medical University, Harbin, China; ^2^School of Public Health, Harbin Medical University, Harbin, China

**Keywords:** socioeconomic strata, PIR, cancer, prevalence, NHANES

## Abstract

**Background:**

Inequality in health outcomes in relation to Americans' socioeconomic status (SES) is rising. American Cancer Society depicts that the most common cancers are diagnosed in men and women in 2021. We aim to study socioeconomic inequalities in related cancers to investigate whether the cancer prevalence differs within the family income to poverty ratio (PIR).

**Methods:**

The study investigated data from adults aged 20–85 years participated in the 1999–2018 National Health and Nutrition Examination Survey (NHANES) who had complete data available on PIR and cancer or malignancy information (*n* = 49,720). Participants were stratified into 3 categories of PIR: high income (PIR ≥ 4), middle income (>1 and <4), or at or below the federal poverty level (≤1).

**Results:**

The prevalence of prostate cancer was higher in the middle-income (3.61% [*n* = 464]) and high-income groups (3.36% [*n* = 227]) than in the low-income group (1.83% [*n* = 84], all *p* < 0.001). The prevalence of breast cancer was higher in middle-income (2.86% [*n* = 390]) and high-income participants (3.48% [*n* = 218]) than in low-income participants (2.00% [*n* = 117], all *p* < 0.001). Compared with the low-income group in men (0.48% [*n* = 22]), a higher prevalence of colon and rectum cancer occurs in the middle-income (0.87% [*n* = 112], *p* = 0.012) and high-income groups (0.89% [*n* = 58], *p* = 0.018). The prevalence of lung cancer in women was lower in high-income participants than middle-income participants (0.10% [*n* = 6] vs. 0.29% [*n* = 39], *p* = 0.014).

**Conclusions:**

Increasing disparities in cancer prevalence were identified across all socioeconomic categories analyzed in this study. To ensure the sustainable development goals, it is a global health priority to understand inequalities in health and to target interventions accordingly.

## Introduction

Cancer is the second leading cause of death in the United States and is a major public health concern throughout the US. It was estimated in 2021 that 1,898,160 new cancer cases would be diagnosed and 608,570 cancer deaths are projected to occur in the US (1). According to the American Cancer Society and the National Cancer Institute, there will be an estimated 22.1 million cancer cases by 2030 (2). Some studies have focused on the role of socioeconomic status (SES) in the formation of cancer mortality and survival (3–11), while the relationship between SES and cancer prevalence received little attention.

Inequalities in accessing health services have been attributed to disparities in SES. Income, along with education level and occupational status, is considered to be a core component of SES. Significant socioeconomic disparities remain in cancer outcomes despite advances in screening, early detection, and cancer treatments. These differences are due in part to unequal access to high-quality, timely cancer care among socioeconomically diverse. Delays in diagnosis and treatment of cancer result in more advanced disease stage at presentation, reducing treatment response rates and worsening prognosis. It is important to determine whether these socioeconomic differences exist and assess the trends in cancer prevalence over time.

**Figure 1:**
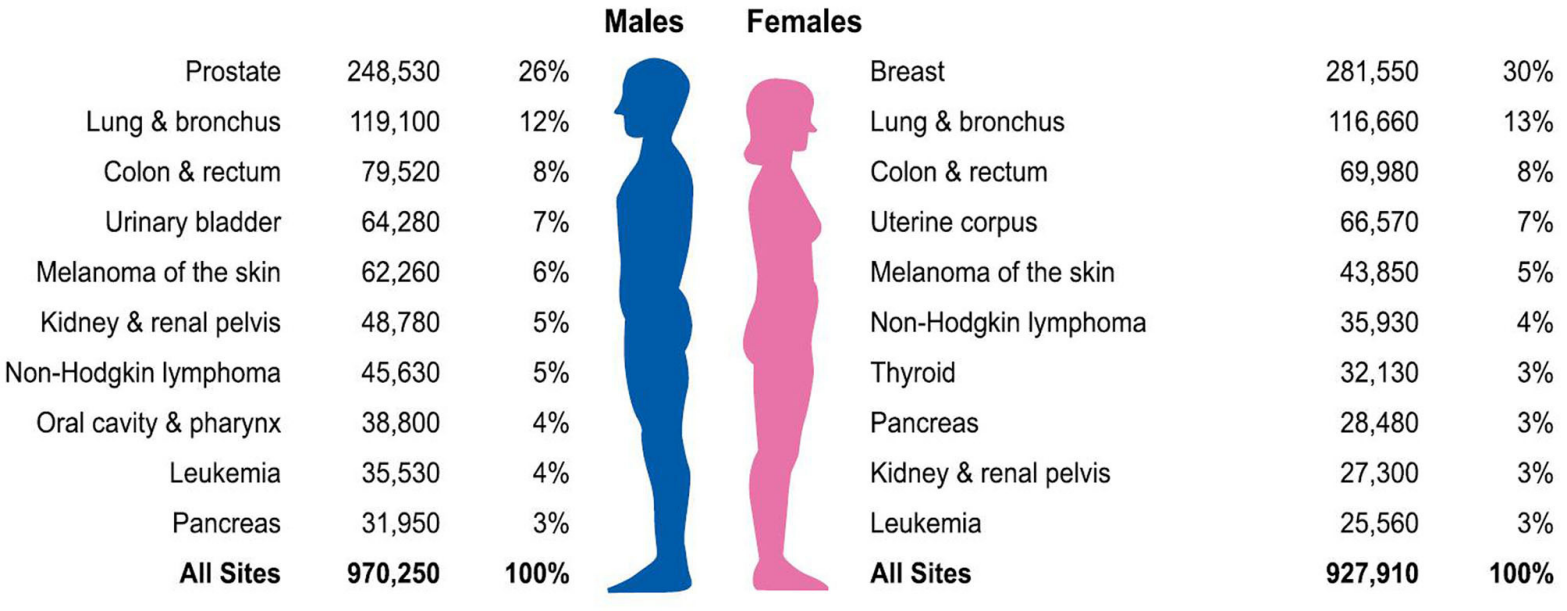
Ten leading cancer types for the estimated new cancer cases by sex, United States, 2021.

Little research has focused on the links between different SES and cancer prevalence, which are dramatically significant. The American Cancer Society describes the most common diagnosis of cancer among men and women in 2021 (1). Prostate cancer, lung cancer, and colon and rectum cancer account for 46% of estimated new cancer cases in men, with prostate cancer alone accounts for 26% of male cancers. For women, breast cancer, lung cancer, and colon and rectum cancer account for 50% of all new diagnoses, with breast cancer alone comprising 30% of diagnoses (Figure 1) (1). Consequently, our objective was to study the association between the abovementioned cancer prevalence and different SES.

## Methods

### Study Population

**Figure 2:**
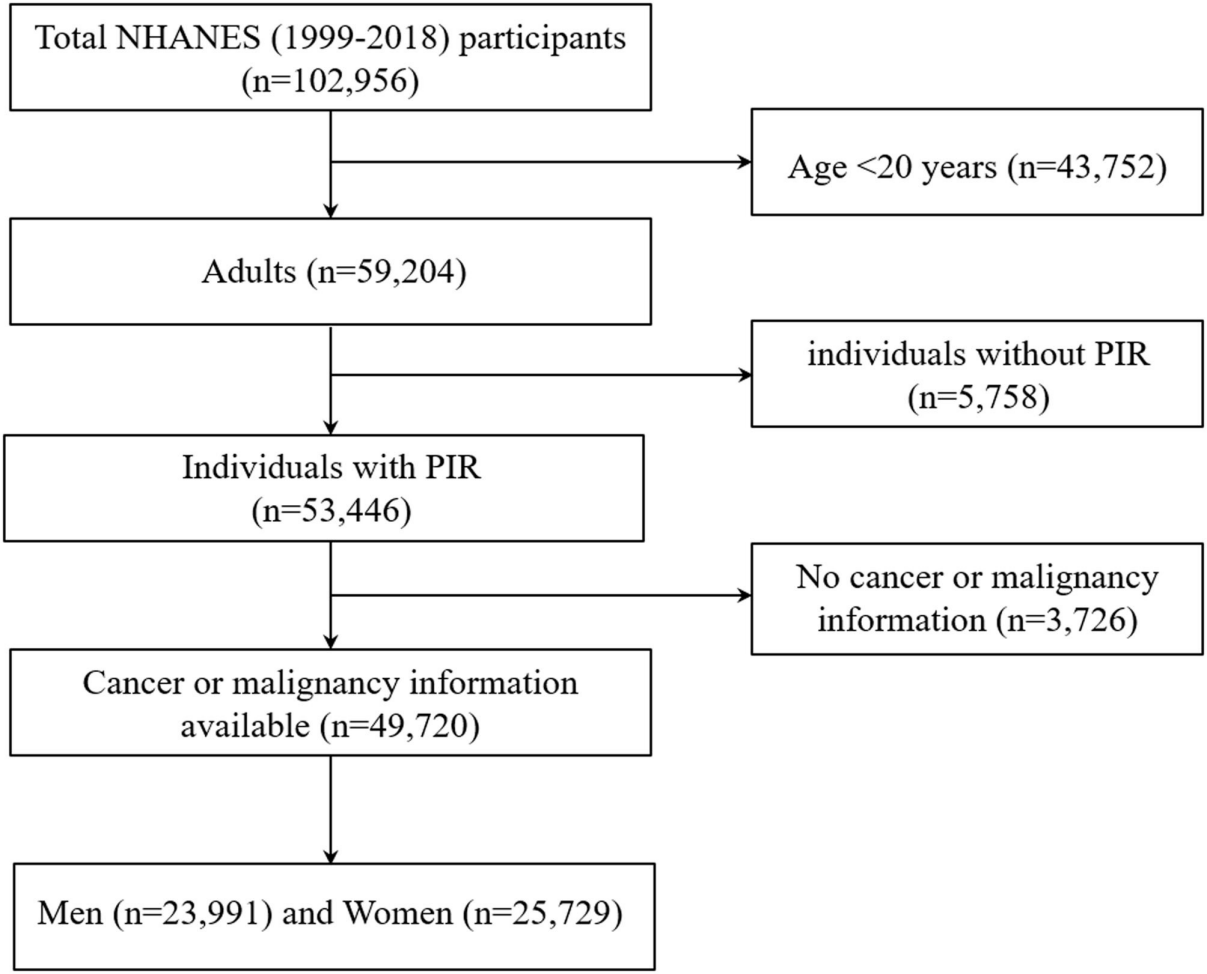
Flow chart of the study population. Describes how the present sample of participants was composed. NHANES, National Health and Nutrition Examination Survey.

The National Health and Nutrition Examination Survey (NHANES) studies are cross-sectional, complex samples of the U.S. civilian non-institutionalized population conducted by the National Center for Health Statistics (NCHS) of the Centers for Disease Control and Prevention (CDC). Additional details on the NHANES survey, sampling methodologies, and design have previously been published (12–15). All participants provided written informed consent and the NCHS institutional review board approved each NHANES cycle. The data from the survey interview and physical examination within continuous NHANES (1999–2018, *n* = 102,956) were analyzed. Our analysis was limited to participants who aged 20 years or older with available malignancy information and family income to poverty ratio (PIR) data (*n* = 49,720; Figure 2).

### Covariant Evaluation

The primary exposure variable was SES, which was assessed based on the PIR. The PIR is a ratio of self-reported household income that accounts for household income according to household or family size, household age composition, and year. Hence, PIR ≤ 1 entails that the household is below the poverty line, and conversely a PIR > 1 entails that the household is above the poverty line. We separated participants into 3 groups: low-income (i.e., at or below the poverty ratio), middle-income (i.e., above the PIR to <4), and high-income (i.e., PIR, ≥ 4) adults. A PIR of ≤1 means that a person is ≤100% below the federal poverty level/threshold. The middle- and high-income groups were assessed by eligibility for subsidies according to the Patient Protection Affordable Care Act (16).

The following variables were self-reported: age, sex, race/ethnicity, marital status, citizenship status, educational level, insurance status, alcohol status, smoking status, physical activity, and family income. Height and weight were measured using standard protocols. Race/ethnicity categories were defined as follows: non-Hispanic white, non-Hispanic black, Mexican American, and other. Body mass index (BMI) (weight in kilograms divided by height in meters squared) was calculated as a BMI of at least 25.0 and obesity as a BMI of at least 30.0. Educational attainment was categorized as less than high school graduate, high school graduate or a general educational development certificate, and greater than high school. Alcohol consumption (none/ <2 drinks per week/≥2 drinks per week), smoking status (non-smoker/former smoker/current smoker), physical activity (none/1–3 times per week/4 or more times per week) were measured through self-reported questionnaires.

### Statistical Analysis

All analyses used the NHANES sampling weights and accounted for the other aspects of the complex survey design. A value of *p* < 0.05 was used as a cutoff to indicate statistical significance. Statistical analysis was performed using both SPSS statistical software (Version 24, IBM corp.) and STATA Statistics/Data Analysis (Version 16.0, Stata Corp.).

Descriptive data are presented as mean ± standard deviation (SD) for continuous variables or numbers and their proportions for categorical variables. Chi-square test statistics were used to examine differences in categorical variables, and ANOVA was used to examine differences in normal continuous variables. The exact chi-square tests with Bonferroni's correction for multiple comparisons were applied to compare the prevalence rates of the cancers between the three groups. To visually illustrate changes in cancer prevalence during consecutive surveys, we calculated the prevalence of each outcome by descriptive statistics.

Multivariate logistic regression was used to determine associations between cancer, demographics, and risk factors. Model 1 included adjustment for age (40–59 vs. 20–39, 60+ vs. 20–39), race/ethnicity (white vs. black, Hispanic/Mexican vs. black, and other vs. black), marital status (married vs. not married), health insurance (covered vs. not covered), an education level (high school or equivalent vs. less than high school, greater than high school vs. less than high school), citizenship status (US citizenship vs. non-US citizenship), and PIR (PIR ≤1.0 vs. PIR 1.0–4.0, PIR ≥4.0 vs. PIR 1.0–4.0); Model 2 adjusted for model 1 variables plus an additional adjustment for BMI (25.0–29.9 vs. <25.0, ≥30.0 vs. <25.0), drinking status (<2 drinks/d vs. non-drinker, ≥2 drinks/d vs. non-drinker), smoking status (former smoker vs. non-smoker, current smoker vs. non-smoker), and physical activity (moderate vs. never, vigorous vs. never).

## Results

In the continuous NHANES (1999–2018) data set, there were 49,720 adults who were 20 years or older and were restricted to the participants whose PIR and cancer or malignancy information were available (Table 1). Clinical characteristics are detailed in Table 1. In men, 18.8% were a low-income group and 27.3% were a high-income group, while in women, 22.7% were a low-income group and 24.3% were a high-income group. Men and women with higher income were more likely to be white, married, have health insurance covered, and had higher educational levels (*p* < 0.001).

**Table 1:** Characteristics of study participants, 1999–2018.

**Characteristics**		**Men PIR**				**Women PIR**			
	**≤1.0** **(*n* = 4581)** **(18.8%)**	**1.0-4.0** **(*n* = 12857)** **(53.6%)**	**≥4.0** **(*n* = 6553)** **(27.3%)**	**P-valve**	**≤1.0** **(*n* = 5844)** **(22.7%)**	**1.0-4.0** **(*n* = 13627)** **(53.0%)**	**≥4.0** **(*n* = 6258)** **(24.3%)**	**P-value**
**Mean (SD) age, y**	47.06 ± 18.41	50.93 ± 18.94	50.58 ± 16.36	<0.001	46.15 ± 18.79	50.57 ± 19.12	49.06 ± 16.11	<0.001
**Race/ethnicity**				<0.001				<0.001
Non-Hispanic white	1461 (31.9%)	5691 (44.3%)	3878 (59.2%)		1757 (30.1%)	6099 (44.8%)	3671 (58.7%)	
Non-Hispanic black	1060 (23.1%)	2748 (21.4%)	1125 (17.2%)		1517 (26%)	2903 (21.3%)	962 (15.4%)	
Mexican American	1180 (25.8%)	2400 (18.7%)	506 (7.7%)		1462 (25%)	2356 (17.3%)	503 (8%)	
Other	880 (19.2%)	2018 (15.7%)	1044 (15.9%)		1108 (19%)	2269 (16.7%)	1122 (17.9%)	
**Marital status**				<0.001				<0.001
Married	1904 (42%)	7275 (57.1%)	4473 (68.9%)		1701 (29.4%)	6341 (47%)	4145 (66.9%)	
Not married	2629 (58%)	5466 (42.9%)	2018 (31.1%)		4084 (70.6%)	7143 (53%)	2052 (33.1%)	
**Health insurance**				<0.001				<0.001
Covered	2703 (59.1%)	9739 (75.8%)	6141 (93.7%)		3949 (67.7%)	11127 (81.7%)	5991 (95.8%)	
Not covered	1871 (40.9%)	3103 (24.2%)	410 (6.3%)		1881 (32.3%)	2493 (18.3%)	263 (4.2%)	
**Education levels**				<0.001				<0.001
Less than high school	2281 (49.9%)	3938 (30.7%)	476 (7.3%)		2723 (46.7%)	3421 (25.1%)	393 (6.3%)	
High school diploma or GED certificate	1091 (23.9%)	3529 (27.5%)	1089 (16.6%)		1381 (23.7%)	3502 (25.7%)	899 (14.4%)	
Greater than high school	1198 (26.2%)	5374 (41.9%)	4987 (76.1%)		1728 (29.6%)	6687 (49.1%)	4964 (79.3%)	
**Citizenship status**				<0.001				<0.001
US citizenship	3411 (74.7%)	11035 (85.9%)	6145 (93.8%)		4503 (77.3%)	11933 (87.7%)	5899 (94.3%)	
Non-US citizenship	1153 (25.3%)	1813 (14.1%)	403 (6.2%)		1320 (22.7%)	1678 (12.3%)	357 (5.7%)	
**BMI, kg/m** ^**2**^				<0.001				<0.001
<25.0	1486 (34.7%)	3412 (28.3%)	1503 (24.3%)		1474 (26.9%)	3735 (29.2%)	2299 (39%)	
25.0-29.9	1544 (36.1%)	4563 (37.9%)	2605 (42.2%)		1517 (27.7%)	3756 (29.4%)	1714 (29.1%)	
≥30.0	1248 (29.2%)	4072 (33.8%)	2065 (33.5%)		2483 (45.4%)	5297 (41.4%)	1887 (32%)	
**Drinking status**				<0.001				<0.001
Non-drinker	384 (12.5%)	941 (10.7%)	311 (6.1%)		1324 (35.4%)	2501 (26.6%)	685 (14.1%)	
<2drinks/d	529 (17.2%)	2080 (23.6%)	1563 (30.7%)		833 (22.3%)	3126 (33.2%)	2098 (43.2%)	
≥2drinks/d	2160 (70.3%)	5803 (65.8%)	3211 (63.1%)		1584 (42.3%)	3781 (40.2%)	2077 (42.7%)	
**Smoking status**				<0.001				<0.001
Non-smoker	1724 (49.6%)	5419 (62.8%)	3451 (78.1%)		3416 (68.9%)	8709 (79.2%)	4204 (87.1%)	
Former smoker	327 (9.4%)	611 (7.1%)	241 (5.5%)		209 (4.2%)	384 (3.5%)	132 (2.7%)	
Current smoker	1428 (41%)	2596 (30.1%)	727 (16.5%)		1332 (26.9%)	1901 (17.3%)	491 (10.2%)	
**Leisure time physical activity**				<0.001				<0.001
Never	2292 (50%)	5687 (44.2%)	1968 (30%)		3522 (60.3%)	6846 (50.2%)	2112 (33.7%)	
Moderate	1035 (22.6%)	3572 (27.8%)	2441 (37.3%)		1146 (19.6%)	3338 (24.5%)	2083 (33.3%)	
Vigorous	1254 (27.4%)	3598 (28%)	2144 (32.7%)		1176 (20.1%)	3443 (25.3%)	2063 (33%)	

*Continuous variables are expressed as mean standard deviation (SD), while classified variables are expressed as numbers and their proportions. We used a chi-square test for classified variables, one-way ANOVA for normal continuous variables, and Kruskar-Wallis test for skewed continuous variables*.

*PIR, family income to poverty ratio; GED, General Educational Development; BMI, body mass index*.

### Prevalence of Cancer by Income Group in men

**Figure 3:**
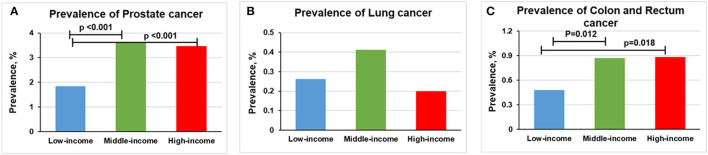
Overall prevalence of cancer among men participants 20 years or older stratified by income group, 1999–2018. Significant at *p* < 0.0167 after Bonferroni correction. **(A)** Prevalence of prostate cancer. **(B)** Prevalence of lung cancer. **(C)** Prevalence of colon and rectum cancer.

The prevalence of prostate cancer was higher in middle-income (3.61% [*n* = 464] vs. 1.83% [*n* = 84], *p* < 0.001) and high-income groups (3.36% [*n* = 227] vs. 1.83% [*n* = 84], *p* < 0.001) than in low-income group (Figure 3A). No statistically significant difference was found between lung cancer and income levels (Figure 3B). Compared with low-income group, a higher prevalence of colon and rectum cancer occurs in middle-income (0.87% [*n* = 112] vs. 0.48% [*n* = 22], *p* = 0.012) and high-income groups (0.89% [*n* = 58] vs. 0.48% [*n* = 22], *p* = 0.018; Figure 3C). However, only middle-income group remained statistically significant (*p* < 0.0167) after Bonferroni correction.

### Prevalence of Cancer by Income Group in Women

**Figure 4:**
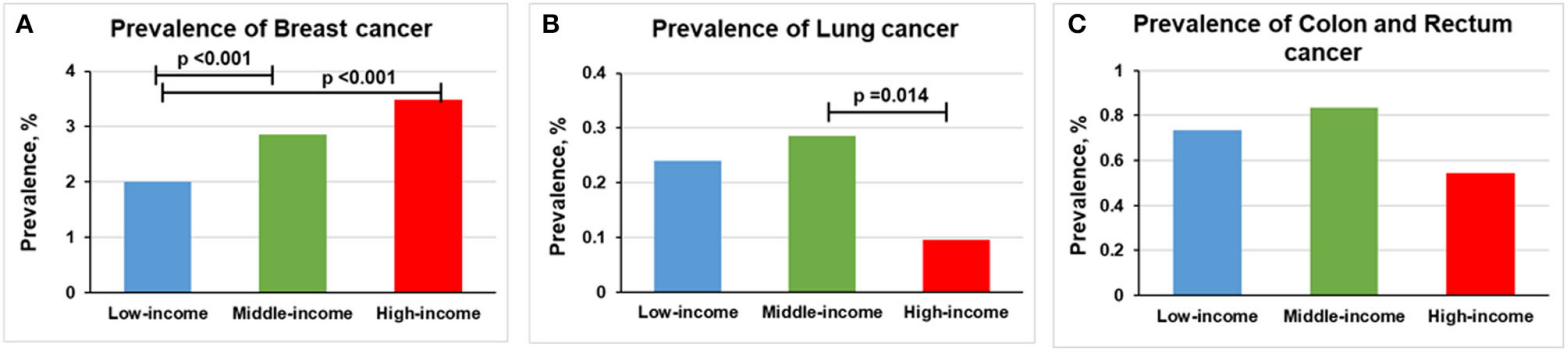
Overall prevalence of cancer among women participants 20 years or older stratified by income group, 1999–2018. Significant at *p* < 0.0167 after Bonferroni correction. **(A)** Prevalence of breast cancer. **(B)** Prevalence of lung cancer. **(C)** Prevalence of colon and rectum cancer.

The prevalence of breast cancer was higher in middle-income (2.86% [*n* = 390] vs. 2.00% [*n* = 117], *p* < 0.001) and high-income participants (3.48% [*n* = 218] vs. 2.00% [*n* = 117], *p* < 0.001) than in low-income participants (Figure 4A). The prevalence of lung cancer was lower in high-income participants than middle-income participants (0.10% [*n* = 6] vs. 0.29% [*n* = 39], *p* = 0.014; Figure 4B). We found no statistically significant relationship between income levels and the prevalence of colon and rectum cancer (Figure 4C).

### Trends for Prevalence of Cancer in men

**Figure 5:**
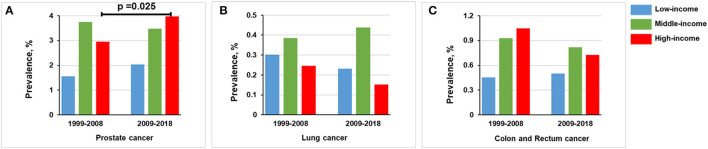
Comparison of prevalence of men in 1999–2008 vs. 2009–2018, stratified by income group. **(A)** Prostate cancer. **(B)** Lung cancer. **(C)** Colon and rectum cancer.

In the high-income group, the prevalence of prostate cancer was increased from 2.952% (*n* = 96) in 1999–2008 to 3.967% (*n* = 131) in 2009–2018 (*p* = 0.025). Lung cancer prevalence was decreased from 0.246% (*n* = 8) in 1999–2008 to 0.151% (*n* = 5) in 2009–2018 (*p* = 0.389). The prevalence of colon and rectum cancer was slightly decreased from 1.046% (*n* = 34) in 1999–2008 to 0.727% (*n* = 24) in 2009–2018 (*p* = 0.168) (Figure 5 and Supplementary Figure S1).

In the middle-income group, the prevalence of prostate cancer in 1999–2008 (3.748%, *n* = 234) is basically the same trend as observed in 2009–2018 (3.477%, *n* = 230; *p* = 0.411). For lung cancer, the prevalence was the same in 1999–2008 (0.384%, *n* = 24) and 2009–2018 (0.438%, *n* = 29; *p* = 0.633). Colon and rectum cancer prevalence was non-significantly decreased from 0.929% (*n* = 58) in 1999–2008 to 0.816% (*n* = 54) in 2009–2018 (*p* = 0.492; Figure 5).

Below the federal poverty level, the prevalence of prostate cancer was increased from 1.562% (*n* = 31) in 1999–2008 to 2.041% (*n* = 53) in 2009–2018 (*p* = 0.232). The prevalence of lung cancer was decreased from 0.302% (*n* = 6) in 1999–2008 to 0.231% (*n* = 6) in 2009–2018 (*p* = 0.640). The prevalence of colon and rectum cancer in 1999–2008 (0.453%, *n* = 9) was comparable to that in 2009–2018 (0.500%, *n* = 13; *p* = 0.820; Figure 5).

### Trends for Prevalence of Cancer in Women

**Figure 6:**
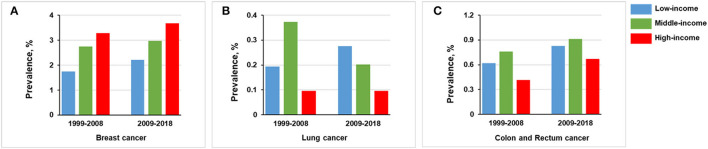
Comparison of prevalence of women in 1999–2008 vs. 2009–2018, stratified by income group. **(A)** Breast cancer. **(B)** Lung cancer. **(C)** Colon and rectum cancer.

For the high-income stratum, the prevalence of breast cancer was increased from 3.289% (*n* = 103) in 1999–2008 to 3.679% (*n* = 115) in 2009–2018 (*p* = 0.400); the prevalence of colon and rectum cancer was increased from 0.415% (*n* = 13) in 1999–2008 to 0.672% (*n* = 21) in 2009–2018 (*p* = 0.167). There were no significant trends for the prevalence of lung cancer between the two groups (*p* = 0.998; Figure 6).

For the middle-income stratum, breast cancer prevalence was increased from 2.743% (*n* = 184) in 1999–2008 to 2.977% (*n* = 206) in 2009–2018 (*p* = 0.414) and colon and rectum cancer from 0.760% (*n* = 51) in 1999–2008 to 0.910% (*n* = 63) in 2018 (*p* = 0.336). In contrast, lung cancer prevalence was decreased from 0.373% (*n* = 25) in 1999–2008 to 0.202% (*n* = 14) in 2009–2018 (*p* = 0.063; Figure 6).

For the low-income stratum, the prevalence of breast cancer was increased from 1.743% (*n* = 45) in 1999–2008 to 2.207 (*n* = 72) in 2009–2018 (*p* = 0.208), lung cancer from 0.194% (*n* = 5) in 1999–2008 to 0.276 % (*n* = 9) in 2009–2018 (*p* = 0.523), and colon and rectum cancer from 0.620% (*n* = 16) in 1999–2008 to 0.828% (*n* = 27) in 2009–2018 (*p* = 0.355; Figure 6).

### The Relationship Between Trends in Income Group and Cancers

Adjusting the models for demographic variables in men, the odds of reporting lung cancer were reduced in the highest resource population over time (odds ratio [OR], 0.533; 95% CI, 0.281–1.013; *p* = 0.055). While, no significant change was observed in prostate cancer (OR, 0.915; 95% CI, 0.765–1.095; *p* = 0.332) or colon and rectum cancer (OR, 1.104; 95% CI, 0.780–1.561; *p* = 0.577; Supplementary Tables S1–S3).

In models adjusted for demographics of women, the high-income group had higher odds of reporting breast cancer (OR, 1.203; 95% CI, 1.001**–**1.446; *p* = 0.049). Conversely, the odds of reporting lung cancer (OR, 0.308; 95% CI, 0.126**–**0.753; *p* = 0.010) and colon and rectum cancer (OR, 0.683; 95% CI, 0.453**–**1.030; *p* = 0.069) were reduced (Supplementary Tables S4–S6). When cancer risk factors were included in the model, the odds of high-income group reporting colon and rectum cancer (OR, 0.225; 95% CI, 0.087**–**0.582; *p* = 0.002) remained low over time, but no statistically significant variation in the odds of reporting breast cancer (OR, 1.105; 95% CI, 0.839**–**1.455; *p* = 0.478) or lung cancer (OR, 0.472; 95% CI, 0.085**–**2.641; *p* = 0.393) (Supplementary Tables S10–S12).

### Association Between Cancer and Other Variables

Both logistic regression analysis models suggest that, in general, older ages tended to be associated with a greater likelihood of reporting cancers. The ORs of cancer ranged from 6.145 (95% CI, 2.558**–**14.763) to 13.072 (95% CI, 1.634**–**104.581) for40**–**59 years age group and from 15.722 (3.749**–**65.930) to 92.179 (12.182**–**697.521) for the oldest age group (60+ years) as compared to the youngest age group (20**–**39 years) (Supplementary Tables S1–S12).

In the fully adjusted model of women (model 2), married vs. non-married individuals had a lower probability of reporting a colon and rectum cancer (OR, 0.567; 95% CI, 0.330**–**0.977; *p* = 0.041). In prostate cancer and breast cancer, health insurance covered vs. not covered participants had higher odds of reporting cancers (OR ranged from 1.912 [95% CI, 1.140**–**3.205] to 3.255 [95% CI, 1.926**–**5.503]) in both model 1 and model 2. Those with US citizenship had a higher probability of reporting breast cancer when compared with those without US citizenship (model 1: OR, 1.591 [95% CI, 1.041**–**2.432]; model 2: OR, 1.859 [95% CI, 1.017**–**3.398]) (Supplementary Tables 1–12).

In the first model of men, as compared to black participants, Hispanic and Mexican participants had a lower probability of reporting prostate cancer (OR, 0.270; 95% CI, 0.188**–**0.388), lung cancer (OR, 0.073; 95% CI, 0.010**–**0.564), and colon and rectum cancer (OR, 0.394; 95% CI, 0.181**–**0.858; Supplementary Tables S1–S3). When cancer risk factors were included in the second model, the odds of Hispanic and Mexican participants reporting prostate cancer (OR, 0.270; 95% CI, 0.135**–**0.541; *p* < 0.001) remained low over time, but no statistically significant variation was reported in the odds of reporting lung cancer (*p* = 0.983) or colon and rectum cancer (*p* = 0.078) (Supplementary Tables S7–S9).

In the first model of women, as compared to black participants, white participants had a higher probability of reporting breast cancer (OR, 1.550; 95% CI, 1.252**–**1.919) and colon and rectum cancer (OR, 1.629; 95% CI, 1.095**–**2.422; Supplementary Tables S4, S6). In a multivariable model adjusted for cancer risk factors (model 2), the association was still statistically significant (OR ranged from 1.849 [95% CI, 1.310**–**2.609] to 2.167 [95% CI, 1.088**–**4.314]) (Supplementary Tables S10, S12).

In the first model, people with a college degree or above are more likely to have prostate cancer (OR, 1.312; 95% CI, 1.079**–**1.596) and breast cancer (OR, 1.293; 95% CI, 1.049**–**1.593) than those without a high school diploma or the General Educational Development (GED) certificate (Supplementary Tables S1, S4). This association no longer reached statistical significance after adjustment for cancer risk factors (Model 2) (Supplementary Tables S7, S10).

## Discussion

### Prostate Cancer

The prevalence of prostate cancer was higher in middle (3.61% [*n* = 464]) and high-income group (3.36% [*n* = 227]) than in low-income group (1.83% [*n* = 84], all *p*s < 0.001). In the high-income group, the prevalence of prostate cancer was increased from 2.952% in 1999–2008 to 3.967% in 2009–2018 (*p* = 0.025). Health insurance covered vs. not covered participants had higher odds of reporting prostate cancer in both model 1 and model 2. People with a college degree or above are more likely to have prostate cancer than those without a high school diploma or GED certificate. Prostate cancer incidence has been robustly correlated with markers of access to care in multiple studies: regions with higher income and educational attainment have higher prostate cancer incidence, which is attributable to increased use of prostate-specific antigen testing (17–20). Prostate carcinoma was positively associated with income, being married, coffee consumption and physical activities from a previously conducted case-control study in Taiwan (21). The results from the National Prostate Cancer Register of Sweden suggested that men with prostate cancer were more often married, educated, and wealthier when compared with men in the control group (22). Accordingly, in low—and middle-income countries, the prognosis of cancer patients is usually poor, because when compared with patients in high-income countries, they have relatively low awareness of cancer, late diagnosis, and lack or unfair access to affordable treatment services (23). These all contribute to a considerable reduction in survival and thus affect prevalence rates.

### Breast Cancer

The prevalence of breast cancer was higher in middle-income (2.86% [*n* = 390]) and high-income participants (3.48% [*n* = 218]) than in low-income participants (2.00% [*n* = 117], all *p*s < 0.001). People with a college degree or above are more likely to have breast cancer than those without a high school diploma or GED certificate. Similarly, a study from the University of Texas MD Anderson Cancer Center showed that participants were relatively highly educated and wealthier than the national average, which may reflect the epidemiology of breast cancer (24). It has also been reported that factors related to low education and low income are a greater obstacle to participating in clinical trials (24, 25). People with a high school degree or lower were less likely to get screened than those with at least a bachelor's degree.

Our study also shows that white participants had a higher probability of reporting breast cancer as compared to black participants in demographic variables adjusted and risk factors adjusted models. Likewise, Rafeek et al. also found that non-white participants were less willing to receive molecular tests or tumor biopsies, even if they were financially secure and used to guide them to use approved drugs for treatment (24). If widespread, these attitudes are likely to lead to a lower detection rate in cancer outcomes.

### Lung Cancer

In our study, after adjusting the models for demographic variables in men, the odds of reporting lung cancer were reduced in the highest resource population over time. We also found that the prevalence of lung cancer in women was lower in high-income participants than middle-income participants in unadjusted and adjusted models. Similarly, a recent paper published by Patel et al. analyzed the incidence trend of lung cancer in California over the past 28 years and found that the increase of female lung adenocarcinoma was more obvious in areas with low SES in the community (26). Consistent with our study, some studies have shown that the risk of lung cancer is negatively correlated with SES factors, such as education, income, and occupation (27–29). SES is related to health status in many ways, such as social resources, physical and psychosocial stressors, and health-related behaviors.

### Colon and Rectum Cancer

Similar findings were reported in our study, when compared with a low-income group of men (0.48%), a higher prevalence of colon and rectum cancer occurs in middle-income (0.87%, *p* = 0.012) and high-income groups (0.89%, *p* = 0.018). Health insurance covered vs. not covered participants had higher odds of reporting colon and rectum cancer in model 1. Indeed, multiple studies have shown that lack of insurance and other socioeconomic factors have been associated with lower colorectal cancer screening rates (30–37). Therefore, when compared with low-income people, high-income people may have more rational health behavior, better understand their symptoms, and better communicate with medical staff. In consequence, the former may have a higher chance of early cancer detection. Based on these considerations, the prevalence of colon and rectum cancer is not the actual prevalence, which could be reasonably considered to be the detection rate. Data from the Behavioral Risk Factor Surveillance System show that the screening rates are persistently low among low-income individuals, people with medical subsidies or no medical insurance, adults with short years of education, ethnic minorities, and residents in rural areas (38, 39). Therefore, we now recognize that the relationship between SES and health may reflect two-way causality (i.e., from better health to higher SES and from higher SES to better health).

Our study has several limitations. First, because this was primarily a cross-sectional study, causality could not be ascertained. Second, the data rely exclusively on self-reported information, which can lead to underreporting or overreporting. In the context of low- and middle income, the reported incidence rate is more susceptible to the bias in the case determination. In the context of high income, the incidence rate of the report is more reliable. However, studies have illustrated that the self-report results of NHANES are reliable and valid (40). Third, the results may not be mature enough due to the rare patients with cancer. These results remained highly interpretable and were still displayed. Further studies investigating this subject and confirming our findings are therefore needed.

## Conclusion

The cross-sectional study found substantial associations between SES disparities that may contribute to differences in prevalence in the United States. Factors, such as health insurance coverage and education levels, also contribute to disparities in cancer prevalence, which are key barriers to accessing cancer screening. Thus, in the long run, policies aimed at socioeconomic inequality may also be an effective mechanism to solve the inequality of cancer prevalence.

## Data Availability Statement

The original contributions presented in the study are included in the article/Supplementary Material, further inquiries can be directed to the corresponding authors.

## Ethics Statement

The studies involving human participants were reviewed and approved by the National Center for Health Statistics Ethics Review Board. The patients/participants provided their written informed consent to participate in this study. Written informed consent was obtained from the individual(s) for the publication of any potentially identifiable images or data included in this article.

## Author Contributions

MW, YLiu, YM, GL, and XZ designed the study. MW, YLi, CS, YC, and PC selected and processed the data. MW wrote the manuscript. GL, XZ, and CS revised the manuscript. All authors contributed to the subsequent drafts, reviewed, and endorsed the final submission.

## Conflict of Interest

The authors declare that the research was conducted in the absence of any commercial or financial relationships that could be construed as a potential conflict of interest.

## Publisher's Note

All claims expressed in this article are solely those of the authors and do not necessarily represent those of their affiliated organizations, or those of the publisher, the editors and the reviewers. Any product that may be evaluated in this article, or claim that may be made by its manufacturer, is not guaranteed or endorsed by the publisher.

## Supplementary Material

Supplementary Figure 1 Trends in prevalence of cancer outcomes among participants 20 years or older stratified by income group, 1999–2018. **(A–C)** Prevalence in men. **(D–F)** Prevalence in women.

Supplementary Table 1 Model 1: Multivariable analysis of the association between prostate cancer and demographics in men, 1999–2018.

Supplementary Table 2 Model 1: Multivariable analysis of the association between lung cancer and demographics in men, 1999–2018.

Supplementary Table 3 Model 1: Multivariable analysis of the association between colon and rectum cancer and demographics in men, 1999–2018.

Supplementary Table 4 Model 1: Multivariable analysis of the association between breast cancer and demographics in women, 1999–2018.

Supplementary Table 5 Model 1: Multivariable analysis of the association between lung cancer and demographics in women, 1999–2018.

Supplementary Table 6 Model 1: Multivariable analysis of the association between colon and rectum cancer and demographics in women, 1999–2018.

Supplementary Table 7 Model 2: Multivariable analysis of the association between prostate cancer, demographics and cancer risk factors in men, 1999–2018.

Supplementary Table 8 Model 2: Multivariable analysis of the association between lung cancer, demographics and cancer risk factors in men, 1999–2018.

Supplementary Table 9 Model 2: Multivariable analysis of the association between colon and rectum cancer, demographics and cancer risk factors in men, 1999–2018.

Supplementary Table 10 Model 2: Multivariable analysis of the association between breast cancer, demographics and cancer risk factors in women, 1999–2018.

Supplementary Table 11 Model 2: Multivariable analysis of the association between lung cancer, demographics and cancer risk factors in women, 1999–2018.

Supplementary Table 12 Model 2: Multivariable analysis of the association between colon and rectum cancer, demographics and cancer risk factors in women, 1999–2018.
