# Supplementary figures and images for: Association Between Cancer Prevalence and Different Socioeconomic Strata in the US: The National Health and Nutrition Examination Survey, 1999–2018
Source: Front Public Health. 2022 Jul 22;10:873805. doi: 10.3389/fpubh.2022.873805 (PMC9355719; doi:10.3389/fpubh.2022.873805)

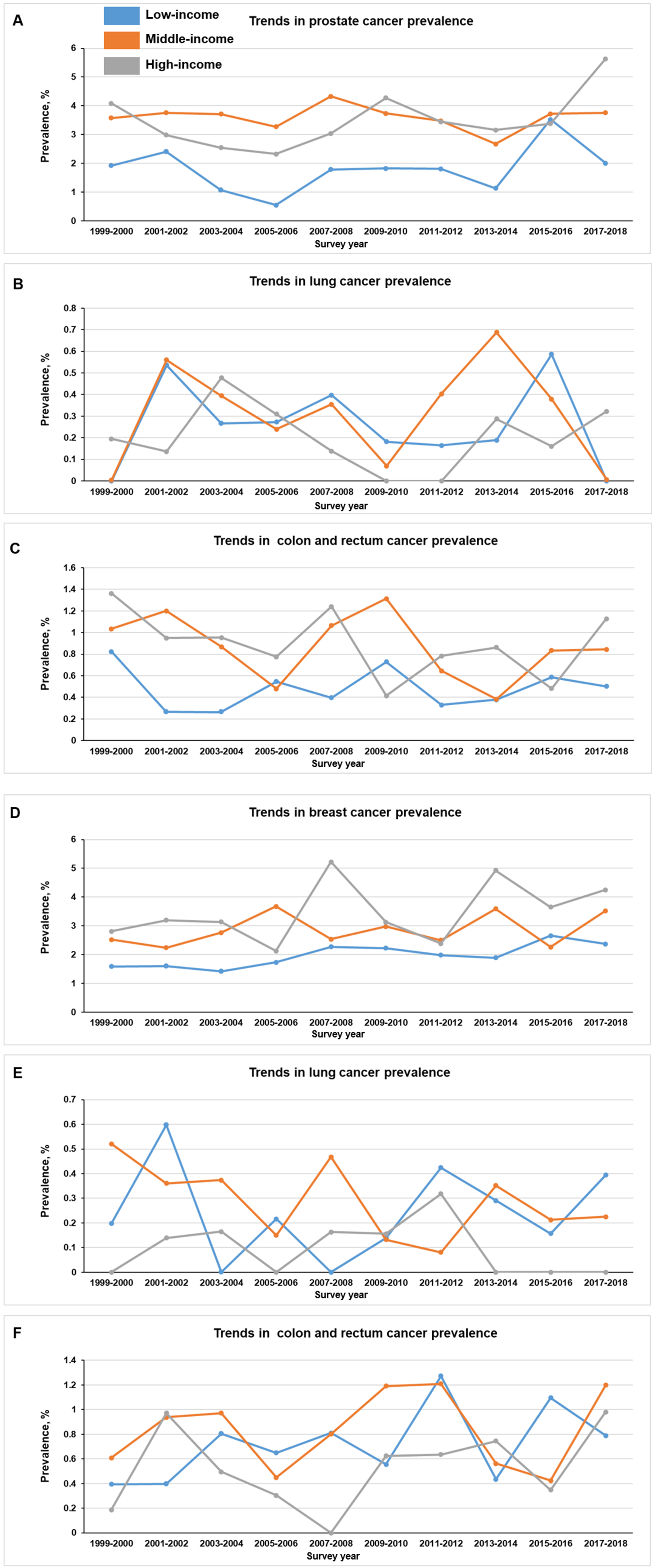

Supplement: Supplementary Figure 1 — Trends in prevalence of cancer outcomes among participants 20 years or older stratified by income group, 1999–2018. (A–C) Prevalence in men. (D–F) Prevalence in women. [file Image_1.TIF]
