# Supplementary material for: Association Between Cancer Prevalence and Different Socioeconomic Strata in the US: The National Health and Nutrition Examination Survey, 1999–2018
Source: Front Public Health. 2022 Jul 22;10:873805. doi: 10.3389/fpubh.2022.873805 (PMC9355719; doi:10.3389/fpubh.2022.873805)
Supplement: Supplementary Table 1 — Model 1: Multivariable analysis of the association between prostate cancer and demographics in men, 1999–2018. [file Table_1.DOCX]

**Supplemental Table 1. Model 1: Multivariable Analysis of the Association Between Prostate Cancer and Demographics in men, 1999-2018.**

| **Variables** | **Odds ratio (95% CI)** | ***p* value** |
| --- | --- | --- |
| **Age** |  |  |
| 40-59 vs. 20-39 | 7100813.777(0.000) | 0.971 |
| 60+ vs. 20-39 | 108209945.868(0.000) | 0.966 |
| **Race/Ethnicity** |  |  |
| White vs. Black | 0.678(0.568-0.809) | 0.000 |
| Hispanic/Mexican vs. Black | 0.270(0.188-0.388) | 0.000 |
| Other vs. Black | 0.466(0.349-0.622) | 0.000 |
| **Marital status** |  |  |
| Married vs. Not married | 1.119(0.946-1.322) | 0.189 |
| **Health insurance** |  |  |
| Covered vs. Not covered | 3.255(1.926-5.503) | 0.000 |
| **Education levels** |  |  |
| High school or equivalent vs. Less than high school | 1.045(0.839-1.301) | 0.692 |
| Greater than high school vs. Less than high school | 1.312(1.079-1.596) | 0.007 |
| **Citizenship status** |  |  |
| US citizenship vs. Non-US citizenship | 1.610(0.978-2.651) | 0.061 |
| **Family income to poverty ratio** |  |  |
| PIR ≤1.0 vs. PIR 1.0-4.0 | 0.797(0.620-1.024) | 0.076 |
| PIR ≥4.0 vs. PIR 1.0-4.0 | 0.915(0.765-1.095) | 0.332 |

Data are from the National Health and Nutrition Examination Survey (NHANES) between 1999-2018 (N=23,991). Marital status missing from 226 participants, health insurance missing from 24 participants, education missing from 28 participants, citizenship status missing from 31 participants.

Akaike Information Criterion (AIC) =5311.367, Bayesian Information Criterion (BIC)=5403.358

**Supplemental Table 2. Model 1: Multivariable Analysis of the Association Between** **Lung Cancer and Demographics in men, 1999-2018.**

| **Variables** | **Odds ratio (95% CI)** | ***p* value** |
| --- | --- | --- |
| **Age** |  |  |
| 40-59 vs. 20-39 | 11.846(1.521-92.291) | 0.018 |
| 60+ vs. 20-39 | 52.706(7.159-388.047) | 0.000 |
| **Race/Ethnicity** |  |  |
| White vs. Black | 1.025(0.591-1.777) | 0.930 |
| Hispanic/Mexican vs. Black | 0.073(0.010-0.564) | 0.012 |
| Other vs. Black | 0.936(0.430-2.040) | 0.868 |
| **Marital status** |  |  |
| Married vs. Not married | 0.830(0.515-1.339) | 0.446 |
| **Health insurance** |  |  |
| Covered vs. Not covered | 1.392(0.535-3.621) | 0.498 |
| **Education levels** |  |  |
| High school or equivalent vs. Less than high school | 0.868(0.480-1.571) | 0.640 |
| Greater than high school vs. Less than high school | 0.744(0.424-1.307) | 0.303 |
| **Citizenship status** |  |  |
| US citizenship vs. Non-US citizenship | 1.209( 0.347 4.206) | 0.766 |
| **Family income to poverty ratio** |  |  |
| PIR ≤1.0 vs. PIR 1.0-4.0 | 0.845(0.438-1.630) | 0.615 |
| PIR ≥4.0 vs. PIR 1.0-4.0 | 0.533(0.281-1.013) | 0.055 |

Data are from the National Health and Nutrition Examination Survey (NHANES) between 1999-2018 (N=23,991). Marital status missing from 226 participants, health insurance missing from 24 participants, education missing from 28 participants, citizenship status missing from 31 participants.

AIC=945.5989, BIC=1050.545

**Supplemental Table 3. Model 1: Multivariable Analysis of the Association Between Colon and Rectum Cancer and Demographics in men, 1999-2018.**

| **Variables** | **Odds ratio (95% CI)** | ***p* value** |
| --- | --- | --- |
| **Age** |  |  |
| 40-59 vs. 20-39 | 9.432(2.196-40.516) | 0.003 |
| 60+ vs. 20-39 | 59.984(14.719-244.456) | 0.000 |
| **Race/Ethnicity** |  |  |
| White vs. Black | 1.403(0.959-2.053) | 0.081 |
| Hispanic/Mexican vs. Black | 0.394(0.181-0.858) | 0.019 |
| Other vs. Black | 0.912(0.512-1.624) | 0.755 |
| **Marital status** |  |  |
| Married vs. Not married | 0.880(0.645-1.202) | 0.423 |
| **Health insurance** |  |  |
| Covered vs. Not covered | 1.790(0.822-3.901) | 0.143 |
| **Education levels** |  |  |
| High school or equivalent vs. Less than high school | 0.692(0.461-1.039) | 0.076 |
| Greater than high school vs. Less than high school | 0.720(0.500-1.035) | 0.076 |
| **Citizenship status** |  |  |
| US citizenship vs. Non-US citizenship | 9.028(1.232-66.148) | 0.030 |
| **Family income to poverty ratio** |  |  |
| PIR ≤1.0 vs. PIR 1.0-4.0 | 0.801(0.498-1.291) | 0.363 |
| PIR ≥4.0 vs. PIR 1.0-4.0 | 1.104(0.780-1.561) | 0.577 |

Data are from the National Health and Nutrition Examination Survey (NHANES) between 1999-2018 (N=23,991). Marital status missing from 226 participants, health insurance missing from 24 participants, education missing from 28 participants, citizenship status missing from 31 participants.

AIC=1948.467, BIC=2053.414

**Supplemental Table 4. Model 1: Multivariable Analysis of the Association Between Breast Cancer and Demographics in women, 1999-2018.**

| **Variables** | **Odds ratio (95% CI)** | ***p* value** |
| --- | --- | --- |
| **Age** |  |  |
| 40-59 vs. 20-39 | 11.369(6.675-19.363) | 0.000 |
| 60+ vs. 20-39 | 37.724(22.496-63.258) | 0.000 |
| **Race/Ethnicity** |  |  |
| White vs. Black | 1.550(1.252-1.919) | 0.000 |
| Hispanic/Mexican vs. Black | 1.009(0.734-1.387) | 0.957 |
| Other vs. Black | 0.986(0.734-1.326) | 0.927 |
| **Marital status** |  |  |
| Married vs. Not married | 0.914(0.778-1.073) | 0.270 |
| **Health insurance** |  |  |
| Covered vs. Not covered | 2.323(1.565-3.449) | 0.000 |
| **Education levels** |  |  |
| High school or equivalent vs. Less than high school | 1.013(0.803-1.278) | 0.914 |
| Greater than high school vs. Less than high school | 1.293(1.049-1.593) | 0.016 |
| **Citizenship status** |  |  |
| US citizenship vs. Non-US citizenship | 1.591(1.041-2.432) | 0.032 |
| **Family income to poverty ratio** |  |  |
| PIR ≤1.0 vs. PIR 1.0-4.0 | 1.043(0.834-1.306) | 0.711 |
| PIR ≥4.0 vs. PIR 1.0-4.0 | 1.203(1.000-1.446) | 0.050 |

Data are from the National Health and Nutrition Examination Survey (NHANES) between 1999-2018 (N=25,729). Marital status missing from 263 participants, health insurance missing from 25 participants, education missing from 31 participants, citizenship status missing from 39 participants.

AIC=5682.893, BIC=5788.737

**Supplemental Table 5. Model 1: Multivariable Analysis of the Association Between Lung Cancer and Demographics in women, 1999-2018.**

| **Variables** | **Odds ratio (95% CI)** | ***p* value** |
| --- | --- | --- |
| **Age** |  |  |
| 40-59 vs. 20-39 | 9.144(2.085-0.102) | 0.003 |
| 60+ vs. 20-39 | 15.722(3.749-65.930) | 0.000 |
| **Race/Ethnicity** |  |  |
| White vs. Black | 1.771(0.895-3.506) | 0.101 |
| Hispanic/Mexican vs. Black | 0.000(0.000) | 0.979 |
| Other vs. Black | 0.469(0.142-1.552) | 0.215 |
| **Marital status** |  |  |
| Married vs. Not married | 1.048(0.602-1.825) | 0.868 |
| **Health insurance** |  |  |
| Covered vs. Not covered | 7.013(0.949-51.843) | 0.056 |
| **Education levels** |  |  |
| High school or equivalent vs. Less than high school | 1.074(0.530-2.176 | 0.842 |
| Greater than high school vs. Less than high school | 0.907(0.455-1.806) | 0.780 |
| **Citizenship status** |  |  |
| US citizenship vs. Non-US citizenship | 0.794(0.176-3.575) | 0.764 |
| **Family income to poverty ratio** |  |  |
| PIR ≤1.0 vs. PIR 1.0-4.0 | 1.335(0.685-2.603) | 0.396 |
| PIR ≥4.0 vs. PIR 1.0-4.0 | 0.308(0.126-0.753) | 0.010 |

Data are from the National Health and Nutrition Examination Survey (NHANES) between 1999-2018 (N=25,729). Marital status missing from 263 participants, health insurance missing from 25 participants, education missing from 31 participants, citizenship status missing from 39 participants.

AIC=742.9107, BIC=838.4314

**Supplemental Table 6. Model 1: Multivariable Analysis of the Association Between Colon and Rectum Cancer and Demographics in women, 1999-2018.**

| **Variables** | **Odds ratio (95% CI)** | ***p* value** |
| --- | --- | --- |
| **Age** |  |  |
| 40-59 vs. 20-39 | 6.145(2.558-14.763) | 0.000 |
| 60+ vs. 20-39 | 22.143(9.716-50.464) | 0.000 |
| **Race/Ethnicity** |  |  |
| White vs. Black | 1.629(1.095-2.422) | 0.016 |
| Hispanic/Mexican vs. Black | 0.754(0.400-1.422) | 0.384 |
| Other vs. Black | 0.973(0.555-1.705) | 0.924 |
| **Marital status** |  |  |
| Married vs. Not married | 0.777(0.568-1.062) | 0.113 |
| **Health insurance** |  |  |
| Covered vs. Not covered | 3.149(1.364-7.269) | 0.007 |
| **Education levels** |  |  |
| High school or equivalent vs. Less than high school | 0.780(0.519-1.174) | 0.234 |
| Greater than high school vs. Less than high school | 0.922(0.638-1.333) | 0.666 |
| **Citizenship status** |  |  |
| US citizenship vs. Non-US citizenship | 1.894(0.798-4.493) | 0.148 |
| **Family income to poverty ratio** |  |  |
| PIR ≤1.0 vs. PIR 1.0-4.0 | 1.280(0.880-1.860) | 0.197 |
| PIR ≥4.0 vs. PIR 1.0-4.0 | 0.683(0.453-1.030) | 0.069 |

Data are from the National Health and Nutrition Examination Survey (NHANES) between 1999-2018 (N=25,729). Marital status missing from 263 participants, health insurance missing from 25 participants, education missing from 31 participants, citizenship status missing from 39 participants.

AIC= 2009.358, BIC=2115.202

**Supplemental Table 7. Model 2: Multivariable Analysis of the Association Between Prostate Cancer, Demographics and Cancer Risk Factors in men, 1999-2018.**

| **Variables** | **Odds ratio (95% CI)** | ***p* value** |
| --- | --- | --- |
| **Age** |  |  |
| 40-59 vs. 20-39 | 6889994.671(0.000) | 0.976 |
| 60+ vs. 20-39 | 98038230.526(0.000) | 0.972 |
| **Race/Ethnicity** |  |  |
| White vs. Black | 0.716(0.513-0.999) | 0.049 |
| Hispanic/Mexican vs. Black | 0.270(0.135-0.541) | 0.000 |
| Other vs. Black | 0.539(0.325-0.892) | 0.016 |
| **Marital status** |  |  |
| Married vs. Not married | 1.029(0.753-1.407) | 0.856 |
| **Health insurance** |  |  |
| Covered vs. Not covered | 3.017(1.305-6.971) | 0.010 |
| **Education levels** |  |  |
| High school or equivalent vs. Less than high school | 1.326(0.867-2.026) | 0.193 |
| Greater than high school vs. Less than high school | 1.288(0.866-1.914) | 0.211 |
| **Citizenship status** |  |  |
| US citizenship vs. Non-US citizenship | 2.591(0.924-7.264) | 0.070 |
| **BMI, kg/m^2^** |  |  |
| 25.0-29.9 vs. <25.0 | 1.021(0.732-1.424) | 0.903 |
| ≥30.0 vs. <25.0 | 0.918(0.634-1.329) | 0.650 |
| **Drinking status** |  |  |
| ＜2drinks/d vs. Non-drinker | 0.557(0.123-2.515) | 0.447 |
| ≥2drinks/d vs. Non-drinker | 0.494(0.113-2.151) | 0.347 |
| **Smoking status** |  |  |
| Former smoker vs. Non-smoker | 1.195(0.662-2.157) | 0.555 |
| Current smoker vs. Non-smoker | 0.541(0.359-0.814) | 0.003 |
| **Leisure time physical activity** |  |  |
| Moderate vs. Never | 1.919(0.455-8.105) | 0.375 |
| Vigorous vs. Never | 1.923(0.444-8.333) | 0.382 |
| **Family income to poverty ratio** |  |  |
| PIR ≤1.0 vs. PIR 1.0-4.0 | 0.933(0.598-1.457) | 0.761 |
| PIR ≥4.0 vs. PIR 1.0-4.0 | 0.854(0.617-1.182) | 0.340 |

Data are from the National Health and Nutrition Examination Survey (NHANES) between 1999-2018 (N=23,991). Marital status missing from 226 participants, health insurance missing from 24 participants, education missing from 28 participants, citizenship status missing from 31 participants, BMI missing from 1,493 participants, drinking status missing from 7,009 participants, smoking status missing from 7,467 participants.

AIC=1670.043, BIC=1805.781

**Supplemental Table 8. Model 2: Multivariable Analysis of the Association Between Lung Cancer, Demographics and Cancer Risk Factors in men, 1999-2018.**

| **Variables** | **Odds ratio (95% CI)** | ***p* value** |
| --- | --- | --- |
| **Age** |  |  |
| 40-59 vs. 20-39 | 348062.607(0.000) | 0.976 |
| 60+ vs. 20-39 | 1907885.087(0.000) | 0.973 |
| **Race/Ethnicity** |  |  |
| White vs. Black | 1.417(0.301-6.675) | 0.659 |
| Hispanic/Mexican vs. Black | 0.000(0.000) | 0.983 |
| Other vs. Black | 0.000(0.000) | 0.983 |
| **Marital status** |  |  |
| Married vs. Not married | 1.596(0.320-7.955) | 0.568 |
| **Health insurance** |  |  |
| Covered vs. Not covered | 494948.641(0.000) | 0.980 |
| **Education levels** |  |  |
| High school or equivalent vs. Less than high school | 0.629(0.107-3.715) | 0.609 |
| Greater than high school vs. Less than high school | 0.413(0.062-2.753) | 0.361 |
| **Citizenship status** |  |  |
| US citizenship vs. Non-US citizenship | 21260.060(0.000) | 0.986 |
| **BMI, kg/m^2^** |  |  |
| 25.0-29.9 vs. <25.0 | 0.316(0.032-3.160) | 0.327 |
| ≥30.0 vs. <25.0 | 1.682(0.352-8.032) | 0.515 |
| **Drinking status** |  |  |
| ＜2drinks/d vs. Non-drinker | 1217814.918(0.000) | 0.987 |
| ≥2drinks/d vs. Non-drinker | 1745326.486(0.000) | 0.987 |
| **Smoking status** |  |  |
| Former smoker vs. Non-smoker | 0.000(0.000) | 0.990 |
| Current smoker vs. Non-smoker | 3.837(0.622-23.667) | 0.147 |
| **Leisure time physical activity** |  |  |
| Moderate vs. Never | 0.335(0.030-3.680) | 0.371 |
| Vigorous vs. Never | 0.544(0.047-6.313) | 0.626 |
| **Family income to poverty ratio** |  |  |
| PIR ≤1.0 vs. PIR 1.0-4.0 | 4.871(0.932-25.454) | 0.061 |
| PIR ≥4.0 vs. PIR 1.0-4.0 | 0.751(0.067-8.457) | 0.817 |

Data are from the National Health and Nutrition Examination Survey (NHANES) between 1999-2018 (N=23,991). Marital status missing from 226 participants, health insurance missing from 24 participants, education missing from 28 participants, citizenship status missing from 31 participants, BMI missing from 1,493 participants, drinking status missing from 7,009 participants, smoking status missing from 7,467 participants.

AIC=119.2488, BIC=203.8645

**Supplemental Table 9. Model 2: Multivariable Analysis of the Association Between Colon and Rectum Cancer, Demographics and Cancer Risk Factors in men, 1999-2018.**

| **Variables** | **Odds ratio (95% CI)** | ***p* value** |
| --- | --- | --- |
| **Age** |  |  |
| 40-59 vs. 20-39 | 13.072(1.634-104.581) | 0.015 |
| 60+ vs. 20-39 | 92.179(12.182-697.521) | 0.000 |
| **Race/Ethnicity** |  |  |
| White vs. Black | 0.907(0.474-1.738) | 0.769 |
| Hispanic/Mexican vs. Black | 0.259(0.057-1.165) | 0.078 |
| Other vs. Black | 0.614(0.218-1.732) | 0.357 |
| **Marital status** |  |  |
| Married vs. Not married | 0.567(0.314-1.022) | 0.059 |
| **Health insurance** |  |  |
| Covered vs. Not covered | 0.846(0.336-2.128) | 0.722 |
| **Education levels** |  |  |
| High school or equivalent vs. Less than high school | 0.511(0.213-1.229) | 0.134 |
| Greater than high school vs. Less than high school | 0.837(0.413-1.696) | 0.621 |
| **Citizenship status** |  |  |
| US citizenship vs. Non-US citizenship | 2139723.594(0.000) | 0.986 |
| **BMI, kg/m^2^** |  |  |
| 25.0-29.9 vs. <25.0 | 1.210(0.595-2.461) | 0.599 |
| ≥30.0 vs. <25.0 | 1.584(0.765-3.282) | 0.216 |
| **Drinking status** |  |  |
| ＜2drinks/d vs. Non-drinker | 9.893(2.102-46.563) | 0.004 |
| ≥2drinks/d vs. Non-drinker | 3.253(0.803-13.170) | 0.098 |
| **Smoking status** |  |  |
| Former smoker vs. Non-smoker | 1.041(0.300-3.616) | 0.949 |
| Current smoker vs. Non-smoker | 0.870(0.423-1.790) | 0.706 |
| **Leisure time physical activity** |  |  |
| Moderate vs. Never | 0.357(0.112-1.139) | 0.082 |
| Vigorous vs. Never | 0.175(0.047-0.655) | 0.010 |
| **Family income to poverty ratio** |  |  |
| PIR ≤1.0 vs. PIR 1.0-4.0 | 0.889(0.390-2.027) | 0.779 |
| PIR ≥4.0 vs. PIR 1.0-4.0 | 0.902(0.458-1.776) | 0.766 |

Data are from the National Health and Nutrition Examination Survey (NHANES) between 1999-2018 (N=23,991). Marital status missing from 226 participants, health insurance missing from 24 participants, education missing from 28 participants, citizenship status missing from 31 participants, BMI missing from 1,493 participants, drinking status missing from 7,009 participants, smoking status missing from 7,467 participants.

AIC=589.6062, BIC=734.2267

**Supplemental Table 10. Model 2: Multivariable Analysis of the Association Between Breast Cancer, Demographics and Cancer Risk Factors in women, 1999-2018.**

| **Variables** | **Odds ratio (95% CI)** | ***p* value** |
| --- | --- | --- |
| **Age** |  |  |
| 40-59 vs. 20-39 | 12.792(6.134-26.676) | 0.000 |
| 60+ vs. 20-39 | 47.939(23.312-98.581) | 0.000 |
| **Race/Ethnicity** |  |  |
| White vs. Black | 1.849(1.310-2.609) | 0.000 |
| Hispanic/Mexican vs. Black | 0.954(0.578-1.573) | 0.853 |
| Other vs. Black | 1.296(0.842-1.995) | 0.239 |
| **Marital status** |  |  |
| Married vs. Not married | 0.903(0.711-1.148) | 0.407 |
| **Health insurance** |  |  |
| Covered vs. Not covered | 1.912(1.140-3.205) | 0.014 |
| **Education levels** |  |  |
| High school or equivalent vs. Less than high school | 0.907(0.628-1.310) | 0.602 |
| Greater than high school vs. Less than high school | 1.341(0.969-1.854) | 0.076 |
| **Citizenship status** |  |  |
| US citizenship vs. Non-US citizenship | 1.859(1.017-3.398) | 0.044 |
| **BMI, kg/m^2^** |  |  |
| 25.0-29.9 vs. <25.0 | 0.924(0.698-1.222) | 0.579 |
| ≥30.0 vs. <25.0 | 0.825(0.624-1.091) | 0.177 |
| **Drinking status** |  |  |
| ＜2drinks/d vs. Non-drinker | 1.431(0.322-6.362) | 0.638 |
| ≥2drinks/d vs. Non-drinker | 1.500(0.351-6.405) | 0.584 |
| **Smoking status** |  |  |
| Former smoker vs. Non-smoker | 1.468(0.770-2.797) | 0.244 |
| Current smoker vs. Non-smoker | 0.874(0.599-1.275) | 0.484 |
| **Leisure time physical activity** |  |  |
| Moderate vs. Never | 0.631(0.149-2.664) | 0.531 |
| Vigorous vs. Never | 0.770(0.176-3.370) | 0.728 |
| **Family income to poverty ratio** |  |  |
| PIR ≤1.0 vs. PIR 1.0-4.0 | 1.320(0.945-1.845) | 0.103 |
| PIR ≥4.0 vs. PIR 1.0-4.0 | 1.105(0.839-1.455) | 0.478 |

Data are from the National Health and Nutrition Examination Survey (NHANES) between 1999-2018 (N=25,729). Marital status missing from 263 participants, health insurance missing from 25 participants, education missing from 31 participants, citizenship status missing from 39 participants, BMI missing from 1,567 participants, drinking status missing from 7,720 participants, smoking status missing from 4,951 participants.

AIC= 2652.454, BIC=2811.555

**Supplemental Table 11. Model 2: Multivariable Analysis of the Association Between Lung Cancer, Demographics and Cancer Risk Factors in women, 1999-2018.**

| **Variables** | **Odds ratio (95% CI)** | ***p* value** |
| --- | --- | --- |
| **Age** |  |  |
| 40-59 vs. 20-39 | 2087120.060(0.000) | 0.974 |
| 60+ vs. 20-39 | 1711505.578(0.000) | 0.974 |
| **Race/Ethnicity** |  |  |
| White vs. Black | 2.514(0.492-12.841) | 0.268 |
| Hispanic/Mexican vs. Black | 0.000(0.000) | 0.983 |
| Other vs. Black | 0.228(0.017-3.048) | 0.264 |
| **Marital status** |  |  |
| Married vs. Not married | 1.327(0.360-4.893) | 0.671 |
| **Health insurance** |  |  |
| Covered vs. Not covered | 2.113(0.243-18.379) | 0.498 |
| **Education levels** |  |  |
| High school or equivalent vs. Less than high school | 0.537(0.046-6.296) | 0.620 |
| Greater than high school vs. Less than high school | 2.585(0.455-14.692) | 0.284 |
| **Citizenship status** |  |  |
| US citizenship vs. Non-US citizenship | 0.172(0.028-1.055) | 0.057 |
| **BMI, kg/m^2^** |  |  |
| 25.0-29.9 vs. <25.0 | 1.163(0.251-5.389) | 0.847 |
| ≥30.0 vs. <25.0 | 0.922(0.197-4.323) | 0.918 |
| **Drinking status** |  |  |
| ＜2drinks/d vs. Non-drinker | 0.000(0.000) | 0.995 |
| ≥2drinks/d vs. Non-drinker | 0.000(0.000) | 0.995 |
| **Smoking status** |  |  |
| Former smoker vs. Non-smoker | 0.000(0.000) | 0.992 |
| Current smoker vs. Non-smoker | 0.335(0.032-3.507) | 0.362 |
| **Leisure time physical activity** |  |  |
| Moderate vs. Never | 76439.044(0.000) | 0.996 |
| Vigorous vs. Never | 586258.327(0.000) | 0.995 |
| **Family income to poverty ratio** |  |  |
| PIR ≤1.0 vs. PIR 1.0-4.0 | 4.132(0.960-17.784) | 0.057 |
| PIR ≥4.0 vs. PIR 1.0-4.0 | 0.472(0.085-2.641) | 0.393 |

Data are from the National Health and Nutrition Examination Survey (NHANES) between 1999-2018 (N=25,729). Marital status missing from 263 participants, health insurance missing from 25 participants, education missing from 31 participants, citizenship status missing from 39 participants, BMI missing from 1,567 participants, drinking status missing from 7,720 participants, smoking status missing from 4,951 participants.

AIC=181.8821, BIC=304.8714

**Supplemental Table 12. Model 2: Multivariable Analysis of the Association Between Colon and Rectum Cancer, Demographics and Cancer Risk Factors in women, 1999-2018.**

| **Variables** | **Odds ratio (95% CI)** | ***p* value** |
| --- | --- | --- |
| **Age** |  |  |
| 40-59 vs. 20-39 | 10.937(2.495-47.947) | 0.002 |
| 60+ vs. 20-39 | 24.638(5.810-104.470) | 0.000 |
| **Race/Ethnicity** |  |  |
| White vs. Black | 2.167(1.088-4.314) | 0.028 |
| Hispanic/Mexican vs. Black | 0.802(0.279-2.309) | 0.683 |
| Other vs. Black | 0.509(0.156-1.653) | 0.261 |
| **Marital status** |  |  |
| Married vs. Not married | 0.567(0.330-0.977) | 0.041 |
| **Health insurance** |  |  |
| Covered vs. Not covered | 3.035(0.911-10.113) | 0.071 |
| **Education levels** |  |  |
| High school or equivalent vs. Less than high school | 0.562(0.266-1.186) | 0.130 |
| Greater than high school vs. Less than high school | 1.002(0.540-1.858) | 0.995 |
| **Citizenship status** |  |  |
| US citizenship vs. Non-US citizenship | 1.401(0.394-4.975) | 0.602 |
| **BMI, kg/m^2^** |  |  |
| 25.0-29.9 vs. <25.0 | 1.428(0.750-2.721) | 0.278 |
| ≥30.0 vs. <25.0 | 1.292(0.681-2.453) | 0.433 |
| **Drinking status** |  |  |
| ＜2drinks/d vs. Non-drinker | 0.000(0.000) | 0.995 |
| ≥2drinks/d vs. Non-drinker | 0.000(0.000) | 0.995 |
| **Smoking status** |  |  |
| Former smoker vs. Non-smoker | 0.000(0.000) | 0.992 |
| Current smoker vs. Non-smoker | 1.565(0.800-3.064) | 0.191 |
| **Leisure time physical activity** |  |  |
| Moderate vs. Never | 2695562.008(0.000) | 0.995 |
| Vigorous vs. Never | 1295743.923(0.000) | 0.995 |
| **Family income to poverty ratio** |  |  |
| PIR ≤1.0 vs. PIR 1.0-4.0 | 1.306(0.702-2.432) | 0.399 |
| PIR ≥4.0 vs. PIR 1.0-4.0 | 0.225(0.087-0.582) | 0.002 |

Data are from the National Health and Nutrition Examination Survey (NHANES) between 1999-2018 (N=25,729). Marital status missing from 263 participants, health insurance missing from 25 participants, education missing from 31 participants, citizenship status missing from 39 participants, BMI missing from 1,567 participants, drinking status missing from 7,720 participants, smoking status missing from 4,951 participants.

AIC=745.9455, BIC=896.7226
